# Examination of patients presenting to the emergency department as “apparently drunk”

**DOI:** 10.1007/s11845-025-03868-w

**Published:** 2025-01-16

**Authors:** Michael Hanrahan, Mary O’Mahony, Darren McLoughlin, Anne Sheahan

**Affiliations:** 1 Department of Public Health – HSE South West (Cork & Kerry), St Finbarr’s Hospital, Douglas Road, Cork, Ireland; 2 Mercy University Hospital, Grenville Place, Cork, Ireland; 3 School of Medicine, University College Cork, Cork, Ireland

**Keywords:** Alcohol, Emergency department, Ireland, Public health, Service evaluation

## Abstract

**Background:**

Alcohol, a significant public health concern, contributes to a substantial burden on emergency services. Identifying avoidable causes of Emergency Department (ED) presentations may reduce hospital overcrowding and benefit public health.

**Aims:**

This prevalence study aims to provide a detailed analysis of patients presenting to the ED at Mercy University Hospital (MUH) as “Apparently Drunk” in 2022 and 2023.

**Methods:**

Data were sourced from the Integrated Patient Management Information System at MUH. All patients recorded as “Apparently Drunk” were included. Data collected included demographics, associated injuries or complaints, mode of arrival, admission status, and discharge status. Descriptive statistics were used to summarise the data and trends were examined by comparing 2022 and 2023 data.

**Results:**

A total of 1662 presentations were categorised as “Apparently Drunk,” representing 3% of all ED visits. There was an 18% increase in such presentations from 2022 to 2023. The majority of patients were male (72%). The median age was 39 years. Notably, 23% of the presentations involved people experiencing homelessness, and 81% were conveyed by ambulance. Significant associated injuries included head injuries (7%), falls/collapses (6%), mental health issues (5%), and assaults (4%).

**Conclusion:**

The findings indicate a growing trend in patients presenting to the ED as “Apparently Drunk”. The high incidence among people experiencing homelessness and the substantial resource utilisation underscores the need for targeted public health interventions and integrated services. Policymakers should consider these findings in the context of potential legislative changes that may impact alcohol availability.

## Introduction

Alcohol consumption is a significant public health challenge globally, and Ireland is no exception. In 2023, Ireland was reported to have one of the highest alcohol consumption rates per capita among OECD countries, with an average consumption of 9.9 L per person [1]. Data from the Health Research Board have identified heavy episodic drinking as a particular concern in Ireland, ranking eighth in the world [2]. Such high levels of alcohol consumption have profound implications for public health and the healthcare system. Excessive alcohol consumption is particularly concerning as it correlates with various health and social issues, including increased emergency department (ED) presentations [2, 3].

Alcohol-related ED presentations place a considerable burden on hospitals and ambulance services [3, 4]. A review of EDs in Ireland performed in 2015/16 revealed that approximately 6% of all presentations were alcohol-related, a figure that can surge to 29% during peak times, such as Sunday mornings [3]. Nearly 60% of these alcohol-related presentations were conveyed by ambulance, highlighting the strain on emergency medical services [3]. Another review of ED presentations in Beaumont Hospital in Dublin in 2021 and 2022 found that alcohol was a factor in one-fifth of ED presentations and one-sixth of hospital admissions [4]. The primary reasons for these presentations include injuries directly associated with alcohol use, severe intoxication, medical conditions resulting from chronic alcohol abuse, and mental health crises exacerbated by alcohol consumption [3, 4].

Given the already significant burden on Ireland’s healthcare system, particularly in the context of severely overcrowded EDs, addressing avoidable alcohol-related presentations is crucial. Reducing these avoidable cases would not only alleviate pressure on emergency services but also improve patient outcomes and overall public health. This study aims to contribute to this goal by providing a detailed analysis of alcohol-related presentations at the Mercy University Hospital (MUH) in Cork.

As an inner-city hospital, MUH faces unique challenges related to alcohol consumption due to its geographical and demographic context. With approximately 220 inpatient beds and an annual ED attendance of 32,000 presentations, optimising the use of hospital resources is essential. In response to a significant number of patients with alcohol-related presentations, the hospital is planning to implement a new service aimed at safer management of alcohol detoxification. To inform this initiative, it is essential to understand recent trends in alcohol-related presentations, the demographic profile of affected patients, associated injuries, and the outcomes of these cases.

Moreover, this descriptive, prevalence study, as part of a wider service evaluation, aims to highlight the broader implications of alcohol policy changes in Ireland. The proposed Sale of Alcohol Bill (2022) by the Irish government, which aims to extend licensing hours and increase the availability of alcohol, poses potential risks. Drawing on evidence from other countries, which links extended alcohol trading hours to increased alcohol-related crime, traffic collisions, and hospital admissions, this study underscores the need for careful consideration of such policy changes [5–11].

By providing a detailed examination of alcohol-related ED presentations and the potential impact of increased alcohol availability, this article aims to inform public health strategies and policy decisions. Ultimately, the goal is to reduce the burden of alcohol-related cases on emergency services and improve public health outcomes in Ireland.

## Methods

### Setting

This descriptive, prevalence study was conducted at the MUH in Cork. Data relating to ED presentations in 2022 and 2023 were analysed in May 2024.

### Data source

Data were extracted from the Integrated Patient Management Information System (IPMS), which records details of ED presentations, admissions and discharges. This system includes fields for documenting presenting complaints as determined at the time of triage. For this study, all patients recorded under the category “Apparently Drunk” were included. This categorisation is one of approximately 70 triage codes available and is used when patients present either clearly intoxicated or with problems likely resulting from alcohol use.

Data collected included demographic information (age, gender, and housing status), details of associated injuries or complaints (e.g., head injuries, falls, assaults, and mental health issues), and additional details including mode of arrival, admission status, and discharge status.

### Inclusion criteria

The inclusion criteria encompassed all patients who presented to the ED in 2022 and 2023 and were coded as “Apparently Drunk” in the IPMS system. This study focused on patients tagged with this specific triage code, acknowledging that the usage and interpretation of this code might vary between triage personnel.

### Analysis

Descriptive statistics were used to summarise the data, using Microsoft Excel and SPSS version 26. Categorical data were presented as counts and percentages and were compared using the Chi-square test. Continuous variables are presented as median and interquartile range (IQR). Comparisons between two groups with continuous data were performed using the Mann–Whitney *U* test and comparisons between three groups used the Kruskal–Wallis test. A *p*-value of less than or equal to 0.05 was considered statistically significant.

The number of “Apparently Drunk” presentations was calculated as a percentage of total ED presentations. Yearly trends were examined by comparing the number of cases from 2022 to 2023 and analysing quarterly data within 2023. Demographic data were analysed to identify the most affected age and gender groups. The proportion of presentations involving people experiencing homelessness was calculated and repeat presentations by the same individuals were noted. For associated injuries, the study calculated the percentage of patients with each type of injury or complaint. Those with a head injury noted at triage were further examined to identify if advanced imaging (i.e., CT brain) was used and if the patient had an intracranial injury. The mode of arrival and outcome of presentations were also analysed to understand the burden on ambulance services and the disposition of patients.

### Ethical considerations

Ethical approval was not sought for this study as this analysis was conducted as part of a health service evaluation. The evaluation utilised anonymised, routinely collected data from the IPMS system.

## Results

### Overview of presentations

Table 1 shows the characteristics and outcomes of patients presenting to the ED as “Apparently Drunk” at MUH in 2022 and 2023. There was an overall increase of 18% in “Apparently Drunk” ED presentations from 2022 to 2023, with a total of 1662 presentations (by 1109 unique patients) in the 2-year period. This represents 3% of the total number of presentations to ED. The majority of the patients were male (72%), with a median age of 39 years. The table also shows that the proportion of patients experiencing homelessness increased by 42% from 2022 to 2023, reaching 25% of the total presentations in 2023. The table also reveals that most of the patients were conveyed by ambulance (82%) and during the night (00:00–05:59 and 18:00–23:59). More patients were admitted to the hospital in 2023 (32, 4%) than in 2022 (19, 3%), with a significant *p*-value of 0.003. The table also indicates that the triage urgency of the patients increased in 2023, with more patients classified as immediate, very urgent or urgent (*p*-value = 0.002). The table also shows that the median time spent in ED decreased by 14% from 2022 to 2023, from 7 to 6 h.

**Table 1:** Overview of patients presenting to the emergency department as “Apparently Drunk” in 2022 and 2023

	2022	2023	Total	Percentage difference	*p*-value
Total	763	899	1662	+ 18%	-
Sex					
Male	543 (71%)	654 (73%)	1197 (72%)	+ 20%	0.474
Female	220 (29%)	245 (27%)	465 (28%)	+ 11%	
Age (median, IQR)	38 (27–51)	40 (28–52)	39 (28–51)	+ 5%	0.079
Age group					
10–19 years	46 (6%)	56 (6%)	102 (6%)	+ 22%	0.630
20–29 years	192 (25%)	195 (22%)	387 (23%)	+ 2%	
30–39 years	186 (24%)	210 (23%)	396 (24%)	+ 11%	
40–49 years	130 (17%)	173 (19%)	303 (18%)	+ 33%	
50–59 years	115 (15%)	155 (17%)	270 (16%)	+ 35%	
60–69 years	61 (8%)	77 (9%)	138 (8%)	+ 26%	
70–79 years	31 (4%)	31 (3%)	62 (4%)	0%	
80–89 years	2 (0.3%)	2 (0.2%)	4 (0.2%)	0%	
People experiencing homelessness	156 (20%)	222 (25%)	378 (23%)	+ 42%	0.039
Time of arrival					
00:00–05:59	346 (45%)	387 (43%)	733 (44%)	+ 12%	0.695
06:00–11:59	28 (4%)	41 (5%)	69 (4%)	+ 46%	
12:00–17:59	105 (14%)	128 (14%)	233 (14%)	+ 22%	
18:00–23:59	284 (37%)	343 (38%)	627 (38%)	+ 21%	
Conveyed by ambulanc**e**	615 (81%)	743 (83%)	1358 (82%)	+ 21%	0.283
Triage urgency					
1–immediate	1 (0.1%)	2 (0.2%)	3 (0.2%)	+ 100%	0.002
2–very urgent	87 (11%)	117 (13%)	204 (12%)	+ 34%	
3–urgent	474 (62%)	606 (67%)	1080 (65%)	+ 28%	
4–standard	140 (18%)	139 (16%)	279 (17%)	− 1%	
5–non-urgent	61 (8%)	35 (4%)	96 (6%)	− 43%	
Outcome					
Admitted	19 (3%)	32 (4%)	51 (3%)	+ 68%	0.003
Discharged	416 (55%)	559 (62%)	975 (59%)	+ 34%	
Left ED before treatment completed	322 (42%)	301 (34%)	623 (38%)	− 7%	
Transferred	6 (1%)	7 (1%)	13 (1%)	+ 17%	
Time spent in ED; hours (median, IQR)	7 (4–10)	6 (4–10)	6 (4–10)	− 14%	0.987
Time spent in ED					
0–2.9 h	138 (18%)	144 (16%)	282 (17%)	+ 4%	0.496
3–5.9 h	209 (27%)	275 (31%)	484 (29%)	+ 32%	
6–11.9 h	290 (38%)	326 (36%)	616 (37%)	+ 12%	
12–23.9 h	99 (13%)	126 (14%)	225 (14%)	+ 27%	
≥ 24 h	27 (4%)	28 (3%)	28 (3%)	+ 4%	

**Fig. 1:**
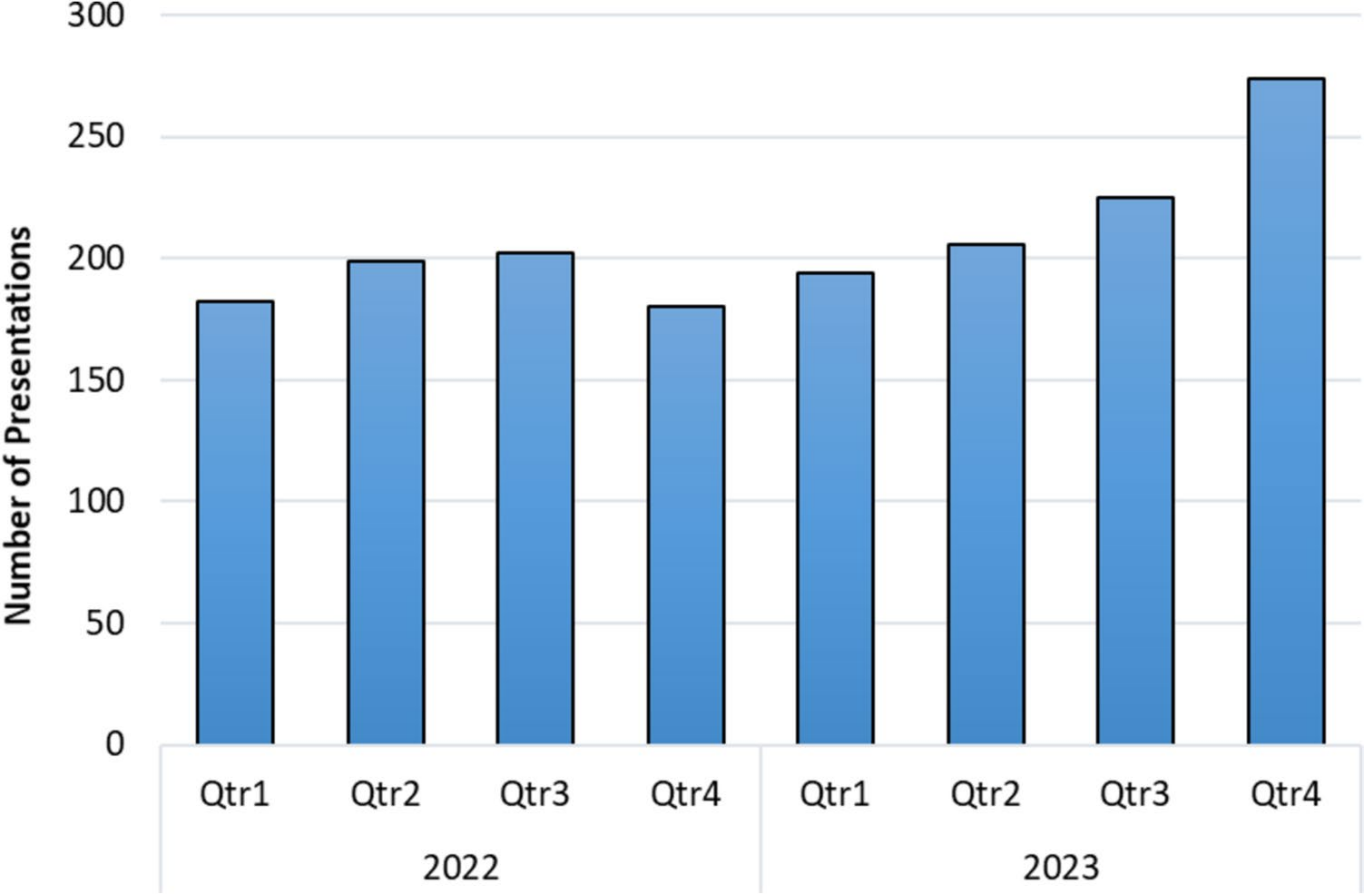
Number of patients presenting to the emergency department as “Apparently Drunk” in 2022 and 2023 per quarter

Examination of the number of presentations per quarter in 2022 and 2023 indicates a rising trend, with each quarter in 2023 seeing an increase in the number of presentations (Fig. 1).

### Sex differences

Table 2 compares the characteristics and outcomes of male and female patients who presented to the ED as “Apparently Drunk”. It shows that the median age of male patients was 41 years, while that of female patients was 33 years, and that the difference was statistically significant (*p*-value < 0.001). It also shows that female patients were more likely to be in the younger age groups (10–19 and 20–29 years), while male patients were more likely to be in the older age groups (50–59 and 70–79 years). The table also indicates that male patients were more likely to be experiencing homelessness, to be admitted to the hospital, and to leave the ED before treatment was completed, while female patients were more likely to be discharged. There was no significant difference in the mode of arrival or the triage urgency between the two groups.

**Table 2:** Comparison of male and female patients presenting to the emergency department as “Apparently Drunk”

	Male	Female	Total	*p*-value
Total	1197	465	1662	-
Age (median, IQR)	41 (31–54)	33 (22–44)	39 (28–51)	< 0.001
Age group				
10–19 years	40 (3%)	62 (13%)	102 (6%)	< 0.001
20–29 years	243 (20%)	144 (31%)	387 (23%)	
30–39 years	297 (25%)	99 (21%)	396 (24%)	
40–49 years	222 (19%)	81 (17%)	303 (18%)	
50–59 years	224 (18.7%)	46 (10%)	270 (16%)	
60–69 years	108 (9%)	30 (7%)	138 (8%)	
70–79 years	59 (5%)	3 (1%)	62 (4%)	
80–89 years	4 (0.3%)	0	4 (0.2%)	
People experiencing homelessness	330 (28%)	48 (10%)	378 (23%)	< 0.001
Conveyed by ambulance	969 (81%)	389 (84%)	1358 (82%)	0.201
Triage urgency				
1–immediate	3 (0.3%)	0	3 (0.2%)	0.062
2–very urgent	152 (13%)	52 (11%)	204 (12%)	
3–urgent	779 (65%)	301 (65%)	1080 (65%)	
4–standard	186 (16%)	93 (20%)	279 (17%)	
5–non-urgent	77 (6%)	19 (4%)	96 (6%)	
Outcome				
Admitted	43 (4%)	8 (2%)	51 (3%)	0.025
Discharged	678 (57%)	297 (64%)	975 (59%)	
Left ED before treatment completed	466 (39%)	157 (34%)	623 (38%)	
Transferred	10 (1%)	3 (1%)	13 (1%)	

**Fig. 2:**
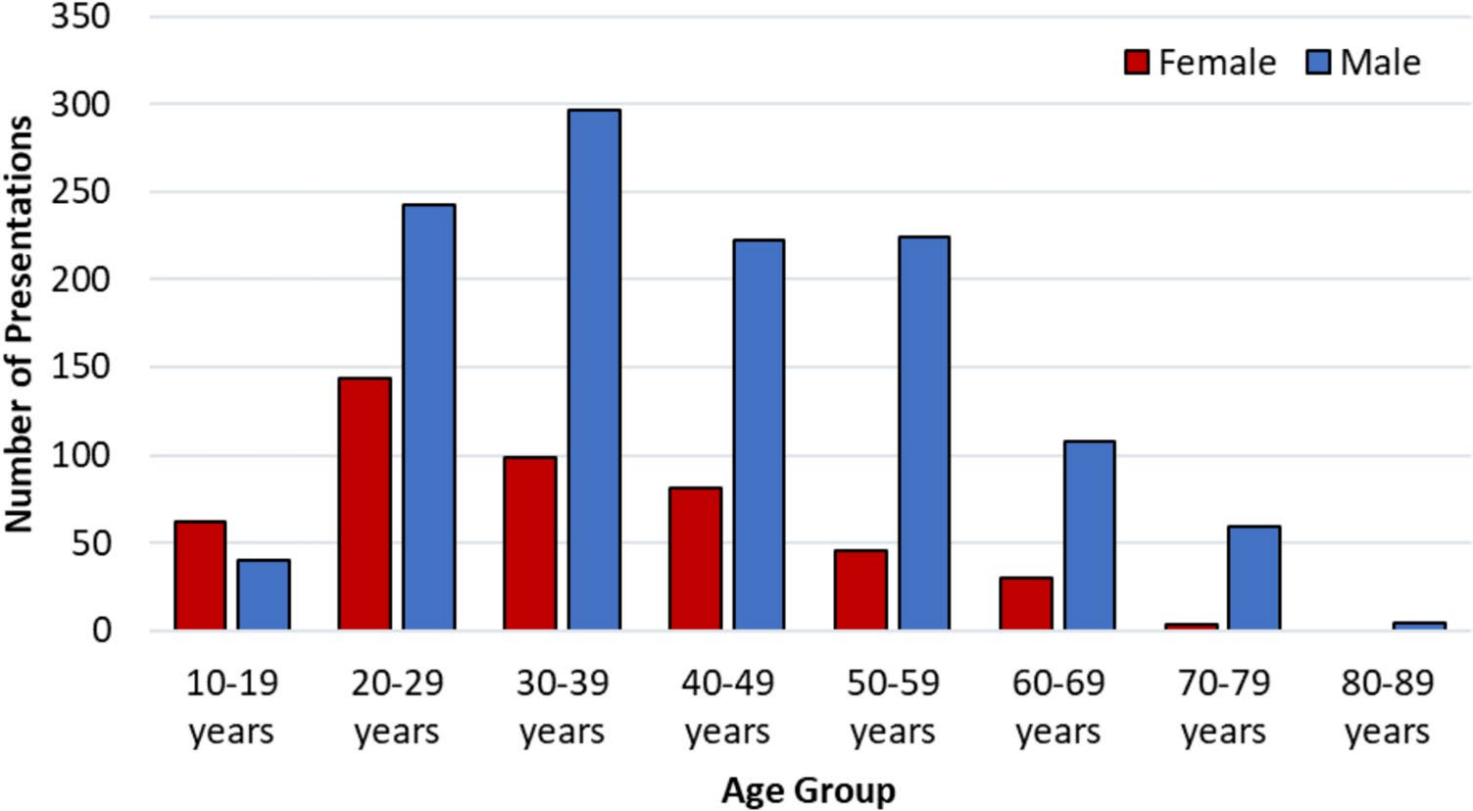
Age and sex of patients presenting to the emergency department as “Apparently Drunk”

Figure 2 illustrates the data by age group and sex. The largest age group among males was 30–39 years, while for females, it was 20–29 years. Notably, the 10- to 19-year age group had more female presentations than male.

### Housing status

Among the patients, 15% were identified as experiencing homelessness. This group accounted for 378 presentations (23% of all “Apparently Drunk” presentations), with 163 individual people experiencing homelessness presenting over the two years. The majority of presentations among people experiencing homelessness were male (87%).

### Time of arrival

**Fig. 3:**
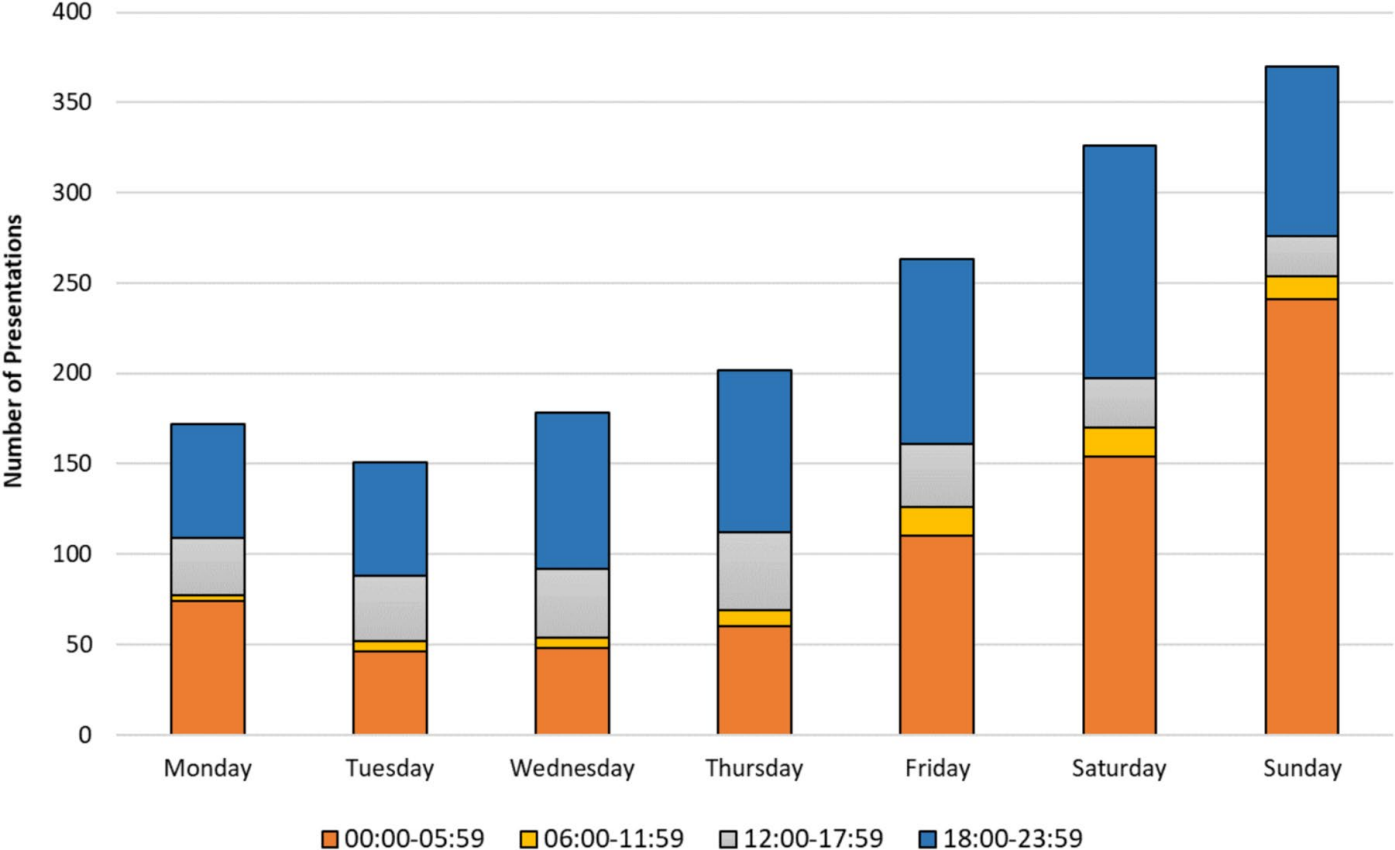
Day and time of patients presenting to the emergency department as “Apparently Drunk”

Figure 3 and Table 3 show the distribution of the time of arrival of the patients who presented to the ED as “Apparently Drunk” by day of the week. The majority of the patients (44%) arrived between 00:00 and 05:59. On Sunday, the proportion arriving at this time was 65%. The lowest number of patients (4%) arrived between 06:00 and 11:59, with no significant difference by day of the week. The table also shows that the peak days for “Apparently Drunk” presentations were Saturdays (20%) and Sundays (22%), while the lowest days were Mondays (10%) and Tuesdays (9%).

**Table 3:** Day and time of patients presenting to the emergency department as “Apparently Drunk”

Time	Monday	Tuesday	Wednesday	Thursday	Friday	Saturday	Sunday	Total
00:00–05:59	74 (43%)	46 (30%)	48 (27%)	60 (30%)	110 (42%)	154 (47%)	241 (65%)	733 (44%)
06:00–11:59	3 (2%)	6 (4%)	6 (3%)	9 (4%)	16 (6%)	16 (5%)	13 (4%)	69 (4%)
12:00–17:59	32 (19%)	36 (24%)	38 (21%)	43 (21%)	35 (13%)	27 (8%)	22 (6%)	233 (14%)
18:00–23:59	63 (37%)	63 (42%)	86 (48%)	90 (45%)	102 (39%)	129 (40%)	94 (25%)	627 (38%)
Total	**172 (10%)**	**151 (9%)**	**178 (11%)**	**202 (12%)**	**263 (16%)**	**326 (20%)**	**370 (22%)**	**1662 (100%)**

### Associated injuries and complaints

Of the 1662 presentations of patients categorised as “Apparently Drunk”, 71% had no additional presenting complaints. However, among those with additional complaints, the following were noted as detailed in Table 4. The table shows that the most common additional presenting complaints were head injury (7%), fall or collapse (6%), mental illness, behaving strangely, or self-harm (5%). The table also compares the frequency of these complaints by sex, showing that males were more likely to have head injuries (8% vs 5%, *p*-value = 0.038) and females were more likely to have falls or collapse (9% vs 5%, *p*-value = 0.004).

**Table 4:** Additional presenting complaints of patients presenting to the ED as “Apparently Drunk”

Presenting complaint	Males (*n* = 1197)	Females (*n* = 465)	Total (*n* = 1662)	*p*-value
Head injury	94 (8%)	23 (5%)	117 (7%)	0.038
Falls/collapse	64 (5%)	43 (9%)	107 (6%)	0.004
Mental illness/behaving strangely/self-harm	61 (5%)	23 (5%)	84 (5%)	0.900
Assault	48 (4%)	14 (3%)	62 (4%)	0.335
Limb problems/wounds/facial problems/eye problems/ear problems	44 (4%)	11 (2%)	55 (3%)	0.180
Abdominal pain/GI bleeding/vomiting and diarrhoea	17 (1%)	6 (1%)	23 (1%)	0.839
Overdose and poisoning	14 (1%)	7 (1%)	21 (1%)	0.582

Of those recorded as having a head injury at triage, 21 (18%) required advanced imaging. Four patients were noted to have had intracranial haemorrhage based on this imaging.

### Outcome

Most patients (59%) were discharged, 38% left the ED before treatment was completed, and 3% were admitted. Fewer patients left the ED before treatment was completed in 2023 (34%) than in 2022 (42%), and more were admitted to the hospital in 2023 (4%) than in 2022 (3%). The difference in admission rates was significant, with a *p*-value of 0.003 (Table 5).

**Table 5:** Comparison of patients presenting to the emergency department as “Apparently Drunk” by outcome (admitted/discharged/left ED before treatment completed)

	Admitted	Discharged	Left ED before treatment completed	*p*-value
Total	**51**	**975**	**623**	**-**
Sex				
Male	43 (84%)	678 (70%)	466 (75%)	0.010
Female	8 (16%)	297 (31%)	157 (25%)	
Age (median, IQR)	52 (41–62)	39 (26–51)	39 (29–51)	0.001
Age group				
10–19 years	0	70 (7%)	30 (5%)	< 0.001
20–29 years	4 (8%)	230 (24%)	148 (24%)	
30–39 years	8 (16%)	225 (23%)	160 (26%)	
40–49 years	11 (22%)	177 (18%)	113 (18%)	
50–59 years	12 (24%)	145 (15%)	113 (18%)	
60–69 years	10 (20%)	87 (9%)	41 (7%)	
70–79 years	6 (12%)	38 (4%)	17 (3%)	
80–89 years	0	3 (0.3%)	1 (0.2%)	
People experiencing homelessness	10 (20%)	214 (22%)	154 (25%)	0.372
Time of arrival				
00:00–05:59	13 (26%)	462 (47%)	252 (40%)	0.001
06:00–11:59	5 (10%)	41 (4%)	22 (4%)	
12:00–17:59	12 (24%)	117 (12%)	103 (17%)	
18:00–23:59	21 (41%)	355 (36%)	246 (40%)	
Conveyed by ambulance	42 (82%)	819 (84%)	489 (79%)	0.020
Triage urgency				
1–immediate	0	2 (0.2%)	0	< 0.001
2–very urgent	13 (26%)	134 (14%)	56 (9%)	
3–urgent	29 (57%)	665 (68%)	375 (60%)	
4–standard	8 (16%)	146 (15%)	125 (20%)	
5–non-urgent	1 (2%)	28 (3%)	67 (11%)	
Time spent in ED; hours (median, IQR)	38 (21–59)	8 (5–11)	5 (3–7)	< 0.001
Time spent in ED				
0–2.9 h	0	86 (9%)	195 (31%)	< 0.001
3–5.9 h	0	255 (26%)	225 (36%)	
6–11.9 h	5 (10%)	431 (44%)	176 (28%)	
12–23.9 h	11 (22%)	187 (19%)	23 (4%)	
≥ 24 h	35 (69%)	16 (2%)	4 (1%)	

Transferred patients are not included

Admitted patients were more likely to present during the daytime compared to those who were discharged or left the department before treatment was completed (34% vs 16% vs 21%, *p*-value = 0.001). Admitted patients were more likely to be triaged as “Very Urgent” compared to discharged patients and those who left the department before treatment was completed (*p*-value < 0.001).

## Discussion

The study underscores the significant impact of alcohol-related presentations on the ED at MUH, emphasizing critical public health concerns. The findings revealed that 1662 presentations, representing 3% of all ED visits over the 2-year period, were categorised as “Apparently Drunk”. This 18% increase from 2022 to 2023 indicates a growing trend that necessitates immediate attention. The majority of these patients were male, with distinct age groups being more affected: males aged 30–39 years and females aged 20–29 years. This demographic trend aligns with existing literature suggesting that young adults and middle-aged individuals are particularly vulnerable to alcohol.

### Implications of findings

The data revealed that 23% of these presentations were from people experiencing homelessness, highlighting a significant intersection between alcohol and housing instability. Frequent presentations by people experiencing homelessness underscore the need for integrated health and social services to address the underlying issues contributing to repeated ED visits. This intersectionality suggests a broader social problem of multiple disadvantages where homelessness and substance abuse are mutually reinforcing issues that require comprehensive and coordinated intervention strategies. Limitations in accessing primary care and other health and social services may also contribute to these ED presentations.

Moreover, there were notable differences in the proportions of people experiencing homelessness, patients with immediate triage urgency, and admitted patients between 2022 and 2023. This suggests a possible increase in the severity and complexity of alcohol-related ED presentations, further straining hospital resources. Part of this observed difference may also be a result of changes in social behaviour and access to or use of alcohol related to the COVID-19 pandemic.

### Associated injuries and concurrent issues

The spectrum of alcohol-related harm is extensive. The data showed that 7% of patients presented with head injuries, 6% with injuries from falls or collapses, 5% had concurrent mental health issues, and 4% reported assaults.

These injuries not only affect the individuals but also place additional burdens on the healthcare system, requiring specialised care and resources. The high prevalence of injuries and mental health issues among these patients calls for targeted interventions that address both physical and mental health needs.

For those with head injuries, 3% are known to have had an intracranial haemorrhage. The majority (82%) of the patients with head injuries were managed clinically and did not require advanced imaging. This places a significant responsibility on the clinicians to ensure they do not expose patients to radiation, unless absolutely necessary. Almost one in five (18%) patients with a head injury noted at triage did have advanced imaging performed. This part of the service evaluation adds to our knowledge of the complexity involved in assessing and managing this challenging cohort of patients. This also has implications for how we may improve our service and the amount of advanced imaging that is required.

### Ambulance utilisation and ED resource burden

The high percentage (81%) of patients conveyed by ambulance underscores the significant strain on emergency medical services. On average, two ambulances per day were dedicated to transporting patients categorised as “Apparently Drunk”, diverting resources from other critical emergencies. This utilisation pattern suggests that alcohol-related incidents are not only a hospital burden but also a substantial operational challenge for emergency medical services.

### Policy implications and future directions

Policies like the Public Health Alcohol Act of 2018, which introduced minimum unit pricing, advertising restrictions and forthcoming health warning labels, represent positive steps towards mitigating alcohol-related harm. However, the study’s findings are particularly pertinent in light of the proposed Sale of Alcohol Bill (2022), which aims to extend alcohol trading hours and increase the availability of alcohol outlets.

International evidence suggests that extended trading hours are associated with increased alcohol-related harm, including higher rates of crime, traffic collisions, domestic violence, and other injuries requiring hospital treatment [6–9]. Additionally, the opposite is true with reductions in alcohol trading hours resulting in less alcohol-related violence [7, 10]. Rural communities may be particularly affected as Norwegian data suggests that extending bar closing hours increases the number of traffic collisions [11].

The potential exacerbation of alcohol-related ED presentations due to increased alcohol availability presents a significant public health risk. Policymakers must weigh the potential benefits of economic gains from extended trading hours against the likely increase in public health and safety risks.

A possible policy intervention that could reduce alcohol-related harm and violence is the introduction of the Cardiff Model, a data monitoring system between hospitals and the police [12]. The Cardiff Model involves the anonymous and routine collection of data on the location, time, and weapon used in violent incidents from patients presenting to the ED [12]. These data are then shared with the police and local authorities to inform crime prevention strategies and target high-risk areas. The Cardiff Model has been shown to reduce violence-related hospital admissions by 35% and serious violence reported by the police by 42% [12]. This is particularly relevant to our study because of the location of MUH in the city centre and the relatively high number of assaults and injuries among our patients. Implementing the Cardiff Model could help reduce the burden of alcohol-related violence on the ED and the community.

### Comparison to other literature

This study reports a comparatively smaller proportion of patients presenting to the ED with an alcohol-related issue compared to other recent Irish literature. McNicholl et al. [3] reported 5.9% of presentations to all EDs in Ireland, and Maharaj et al. [4] reported that 19.4% of presentations to Beaumont ED were alcohol-related. However, when comparing the findings of these studies to this study, it should be noted that the time period is not comparable. McNicholl et al. [3] studied four different 6-h periods selected in December 2015 and January 2016. Maharaj et al. [4] conducted their study over four 1-week periods between 4 pm and 4 am during the 8th to 15th of November, 28th of November to 4th of December 2021, 28th of February to 7th of March, and 3rd to 9th of April 2022. In addition, that study only included patients willing to participate in the survey and had a response rate of 73% [4]. In contrast, this study included all ED presentations to MUH over a 2-year period. As alcohol-related presentations are less frequent during weekdays and during the day, this may partially explain the lower proportion of alcohol-related ED presentations. On the other hand, the data presented may underestimate the true rate of alcohol-related presentations in MUH as the data source used was not designed for this purpose.

### Limitations

Several limitations were acknowledged:

- Data reliability: The reliance on routinely collected data, not originally intended for service improvement analysis, may introduce inaccuracies or missing data.
- Underreporting of alcohol-related presentations: Not all alcohol-related presentations may be captured under the “Apparently Drunk” code, possibly leading to an underestimation of the true burden of alcohol-related cases.
- Variability in triage coding: As the triage code “Apparently Drunk” is applied based on the subjective assessment of triage personnel, its use and interpretation may vary, leading to potential inconsistencies. For example, some patients categorised as “Apparently Drunk” may not be intoxicated at the time of presentation, but this code was applied because the injuries sustained were deemed to be a result of alcohol. Intoxication with other drugs/substances also cannot be excluded. Additionally, associated injuries or co-presenting complaints may not be recorded consistently as only one presenting complaint is required by IPMS, and therefore, it is possible that not all presenting complaints are captured.

### Recommendations

1. Enhanced public health interventions: There is a critical need for targeted public health interventions to reduce alcohol consumption.
2. Reduce alcohol-related violence: Consideration should be given towards implementing the Cardiff Model, or similar, in Cork City. Collaboration and data-sharing between the city council, Gardaí, MUH, and Public Health may enhance the combined response to alcohol-related violence and lead to a safer community.
3. Integrated services for people experiencing homelessness: Given the high proportion of people experiencing homelessness, integrated health and social services should be developed to address both alcohol use and housing instability.
4. Policy considerations: Policymakers should carefully consider the potential public health implications of the Sale of Alcohol Bill (2022) and prioritise policies that mitigate alcohol-related harm.

## Conclusion

This study highlights the significant and growing burden of alcohol-related ED presentations at MUH. The increase in such cases, coupled with substantial resource utilisation and associated injuries, underscores the need for comprehensive public health strategies and cautious policy decisions regarding alcohol availability. Addressing the issue of alcohol through targeted interventions and supportive policies is essential to improving public health outcomes and reducing the strain on emergency services.

By understanding these trends and implementing effective strategies, there is potential to mitigate the adverse effects of alcohol on both individuals and the broader healthcare system. Continued research and policy evaluation are necessary to ensure that interventions remain effective and responsive to the evolving public health landscape.

## References

[1] Doyle A, Mongan D, Galvin B (2024) Alcohol: availability, affordability, related harm, and policy in Ireland. HRB overview series 13. Dublin: Health Research Board

[2] O’Dwyer C, Mongan D, Millar SR et al (2019) Drinking patterns and the distribution of alcohol-related harms in Ireland: evidence for the prevention paradox. BMC Public Health 19:1–9. 10.1186/s12889-019-7666-4 31640654 10.1186/s12889-019-7666-4 PMC6805445

[3] McNicholl B, Goggin D, O’Donovan D (2018) Alcohol-related presentations to emergency departments in Ireland: a descriptive prevalence study. BMJ Open 8:e021932. 10.1136/bmjopen-2018-021932 29794104 10.1136/bmjopen-2018-021932 PMC5988151

[4] Maharaj T, Fitzgerald N, Gilligan E et al (2024) Alcohol-related emergency department presentations and hospital admissions around the time of minimum unit pricing in Ireland. Public Health 227:38–41. 10.1016/j.puhe.2023.11.016 38103275 10.1016/j.puhe.2023.11.016

[5] Burton R, Henn C, Lavoie D et al (2017) A rapid evidence review of the effectiveness and cost-effectiveness of alcohol control policies: an English perspective. Lancet 389(10078):1558–1580. 10.1016/S0140-6736(16)32420-5 27919442 10.1016/S0140-6736(16)32420-5

[6] Rossow I, Norström T (2012) The impact of small changes in bar closing hours on violence. The Norwegian experience from 18 cities. Addiction 107(3):530–7. 10.1111/j.1360-0443.2011.03643.x 21906198 10.1111/j.1360-0443.2011.03643.x PMC3380552

[7] Wilkinson C, Livingston M, Room R (2016) Impacts of changes to trading hours of liquor licences on alcohol-related harm: a systematic review 2005–2015. Public Health Res Pract 26(4):e2641644. 10.26181/22246558.v1 10.17061/phrp2641644 27714387

[8] de Goeij MC, Veldhuizen EM, Buster MC et al (2015) The impact of extended closing times of alcohol outlets on alcohol-related injuries in the nightlife areas of Amsterdam: a controlled before-and-after evaluation. Addiction 110(6):955–964. 10.1111/add.12886 25689068 10.1111/add.12886

[9] Kowalski M, Livingston M, Wilkinson C et al (2023) An overlooked effect: domestic violence and alcohol policies in the night-time economy. Addiction 118(8):1471–1481. 10.1111/add.16192 36967701 10.1111/add.16192 PMC10952798

[10] Deacon JW, Preisz P, Chambers AJ (2022) Sydney ‘lockout’liquor licensing law restrictions have been associated with a sustained reduction in emergency department presentations from assaults over 5 years. Emerg Med Australas 34(5):698–703. 10.1111/1742-6723.13955 35261152 10.1111/1742-6723.13955 PMC9790191

[11] Green C, Krehic L (2022) An extra hour wasted? Bar closing hours and traffic accidents in Norway. Health Econ 31(8):1752–1769. 10.1002/hec.4550 35648106 10.1002/hec.4550 PMC9545209

[12] Florence C, Shepherd J, Brennan I et al (2011) Effectiveness of anonymised information sharing and use in health service, police, and local government partnership for preventing violence related injury: experimental study and time series analysis. Bmj 342. 10.1136/bmj.d3313 10.1136/bmj.d3313 PMC3116927 21680632

